# Carbon balance of plywood from a social reforestation program in Indonesia

**DOI:** 10.1038/s41598-023-40580-0

**Published:** 2023-08-20

**Authors:** Daniel Philipp Müller, Nadine Szemkus, Michael Hiete

**Affiliations:** 1 Department of Business Chemistry, Ulm University, Helmholtzstr. 18, 89081 Ulm, Germany; 2 Study Programme Sustainable Corporate Management, Ulm University, Helmholtzstr. 18, 89081 Ulm, Germany

**Keywords:** Climate-change mitigation, Climate-change policy, Sustainability

## Abstract

Social reforestation programs plant trees on degraded, uncultivated land in low-income regions to allow the local population to generate income from selling wood products and—in case of agroforestry systems—to grow food. For fundraising it is of interest to demonstrate not only positive social impacts but also environmental ones. Proving negative greenhouse gas (GHG) emissions would allow the programs to enter the market for carbon offsetting projects and liberate further funding. In a case study, a social reforestation program in Kalimantan, Indonesia, is analyzed. GHG emissions (according to ISO 14067, PAS 2050 and EU ILCD Handbook for LCA) of the main product, laminated veneer lumber plywood, are determined as 622 and 21 kg CO_2_-e/m^3^ for short-term and long-term (above 100 years) plywood use, respectively. Switching to lignin-based resins and renewable electricity could reduce emissions down to − 363 kg CO_2_-e/m^3^ for long-term use. The analyzed agroforestry system produces almost carbon–neutral plywood today and could be climate positive in the mid-term.

## Introduction

Forests are under pressure worldwide. The global forest area decreased by 45 million hectares between 1992 and 2018, especially in tropical latitudes^1^. Deforestation has among others negative effects on the local and global climate, in particular due to the release of carbon stocks into the atmosphere and alterations of the hydrological cycle, on biodiversity and on soil quality but also on indigenous peoples. Deforestation is a complex problem with a variety of drivers^2^ with the expansion of arable land for producing food, feed, and liquid biofuels (e.g., WWF report^3^) but also fuelwood and timber production as the most important ones. The fast degradation of tropical agricultural soils, when not properly managed, drives fire clearing to get new fertile soils. Further drivers are population growth and, interlinked to it, poverty which is further fueled by climate change leading to failures of crops.

In many developing and newly industrialized countries the rural population often has hardly any opportunities to acquire income. Most are farmers with small, cultivated areas. By logging the local virgin forests, a small income can be generated in addition to the food grown and land for cultivation is generated^4^.

One possible solution to this problem is to make degraded land available to the rural population for agroforestry^5^. Agroforestry systems are able to enhance the food productivity and over all sustainability of forests^6^. Agroforestry has the potential to increase the income of small farmers, but there are many remaining challenges, like the lacking support of governments and lack of access to financial funds for investments in seeds and other needed goods for small farmers^7^. In general, agroforestry systems contribute to farmer incomes^8^, also in regions difficult to plant^9^. In addition, agroforestry sytems often go hand in hand with a legalization of the only tolerated use of state-owned forests by smallholders by officially transferring use rights to the local community, resulting in a social and economic benefit to the locals^10,11^.

Besides the benefits to food security and farmer income, benefits also exist for the entire local community. Many local projects are carried out in partnership between local people, non-profit organizations, local government, and downstream industries, minimizing the risks of social conflicts while generating additional revenue for the local government^12^. Race and Sumirat see further potential improvements through the increase in social capital, extending into sectors other than forestry, such as health, education and agribusiness^13^. For further reading on potentials and hurdles of agroforestry and social forestry in Indonesia, the reviews of Gunawan et al.^14^ and Rakatama and Pandit^15^ also in terms of social and income aspects of the local population as well as impacts on the local community, give an in-depth overview. The benefits for the local population described above are primarily potentials that must also be realized through good cooperation between all parties involved.

Social reforestation programs support the local population with this process, e.g. Fairventure Worldwide (FVW), a non-profit organization based in Germany, by building capacity in agroforestry taking into account the traditional knowledge of the farmers, providing tree seedlings, setting up supply chains for higher-value wood products and establishing a monitoring^16^. Besides reducing the pressure on virgin forests, forests represent a significant natural carbon sink^17,18^ and the carbon sequestration potential of afforestation and reforestation is estimated at 0.5 to 7 Gt CO_2_ annually in 2050^19^. Community forests in Sumatra and Kalimantan, Indonesia, contributed evidentially to the prevention of deforestation^20^, which is beneficial for climate change mitigation. Community forests in Indonesia are a carbon sink with a storage potential of 331 tons C/ha^21^. For organizations such as FVW the carbon sequestration potential of their reforestation projects may be highly important, not only because of mitigating climate change, but also because such organizations depend on donations. An expected or even proven carbon sequestration and the resulting positive climate effects might not only attract new donators but would also allow to take part in the market for carbon offsetting projects with corresponding payments to the reforestation programs. The latter has also resulted in reforestation/afforestation projects which are highly criticized for having negative effects on local communities^17^, e.g. due to land grabbing, on biodiversity because often alien species and monocultures are planted and for having only very low sequestration potentials as a high share of tree seedlings die off. Furthermore, relevant amounts of carbon are sequestrated only after decades whereas certificate holders emit greenhouse gases (GHG) now and the selling of indulgences might prevent that certificate holders cut their emissions.

Several carbon accounting standards have been developed, e.g.,^22–24^. Though they share the common goal of determining the global warming potential (GWP) of a product, the methods slightly differ, in particular with respect to the way biogenic carbon is accounted for. As a consequence, calculated carbon footprints (CF) of the same product can differ considerably (c.f.^64^) for a comparison between DIN EN ISO 14067, PAS 2050 (British Standard Institute), and GHG Protocol (WRI/WBCSD)). The distinction between fossil and biogenic carbon seems trivial but depends on several factors. One factor is the type of forest. ILCD (2010) requires to count carbon emissions from virgin forests and from land use change (LUC) as fossil carbon emissions and emissions from plantation forests as biogenic ones^66^. Further factors are carbon storage time and the distinction between carbon stored directly in the product and stored elsewhere, for example, in the soil. There are approaches that distinguish between short- and long-term storage of carbon. Usually, an assessment period of 100 years is considered. The plywood in this study might achieve a lifetime of more than 100 years if used as structural element in small dwellings for the local residents. Additionally, emissions that occur after the production stage but still in the assessment period, could be calculated as delayed emissions. The added value for an LCA from considering that some emissions take place later is controversial^25^. Further factors include an extension of the time until the wood is oxidized, e.g. due to reuse, recycling and cascade uses, as well as substitution of energy-intensive materials^26^.

This study analyzes an agroforestry project of the Fairventure Worldwide (FVW) organization in Kalimantan, Indonesia, cf.^27^ with respect to its carbon sequestration potential. Within the project, fast-growing sengon tree (*Paraserianthes falcataria *(L.), also called albizia, cf.^28^) seedlings are planted and harvested by the farmers. Sengon trees are adapted to tropical climate, cope with nutrient-poor soils, and are suitable for agroforestry. They grow fast and are typically harvested at an age of 5–7 years. It is an important timber species in Indonesia’s processing industry and often grown in an intercropping approach with agricultural crops^29^. Sengon lightwood has a mean density of 317 kg/m^3^ ^30^ and can be used for furniture and housing construction (cf. the goals of Social Reforestation Program^27,31^). The wood shows a number of searched properties such as a high E-modulus shear strength and breaking strength as well as a high compressive strength important for wall elements in timber construction^32^. The same author reports also that breaking and tensile strengths are disproportionately high in relation to the material density and that the wood is termite resistant and shows a good fire resistance. The wood is sold to local sawmills where it is processed to plywood, an engineered wood product consisting of multiple thin wood layers glued together using adhesives. This plywood can be used by the locals for building affordable and sustainable houses but can be also sold on national and international markets. Recent studies highlight the good mechanical properties of laminated veneer lumber (LVL) made from sengon^33,34^ (LVL resembles plywood with the exception that in LVL veneers all stack in the same direction, whereas in plywood the veneers switch their direction)^35^. Though the adhesives such as phenol–formaldehyde resin only make up around two mass percent of the product, they are responsible for a large share of its GWP^36^. In a systematic literature review, Eisen et al. found that in most studies adhesives with shares of renewable raw materials perform as good as fossil fuel-based ones or even better in environmental terms^78^. A renewable alternative is lignin. It accounts for about 30% of the lignocellulosic biomass^37^ and is a side product in paper making and of lignocellulosic biorefineries. The chemical structure of lignin, a polyphenolic polymer with aldehyde, alcohol, hydroxyl and phenolic hydroxyl groups, makes it particularly suitable for the production of a renewable phenolic resin^38^. Lignin may substitute phenol in phenol formaldehyde resins (e.g.,^39^) but there are also novel adhesive systems made of lignin (e.g.,^40^. Several studies showed a suitable performance of lignin-based resins according to different standards^41,42^. In summary, sengon trees seem appropriate for agroforestry on degraded tropical land and sengon wood shows a number of searched properties. An open question is, however, if reforestation and agroforestry with sengon trees with subsequent production of plywood is also climate positive. The main research question is thus: *How much carbon per m*^*3*^* product is sequestrated if wood from the social afforestation program under study in Indonesia is used in plywood?*

with sub-questions (Q1–Q3):

- *Q1: How large are the GHG emissions occurring during the reforestation and production of plywood?*
- *Q2: How does the use of different carbon accounting standards and use-phase-scenarios affect the assessment of biogenic carbon sequestration?*
- *Q3: What is the potential of substituting fossil-based adhesives with lignin-based adhesives for carbon sequestration?*

## Methods

**Figure 1:**
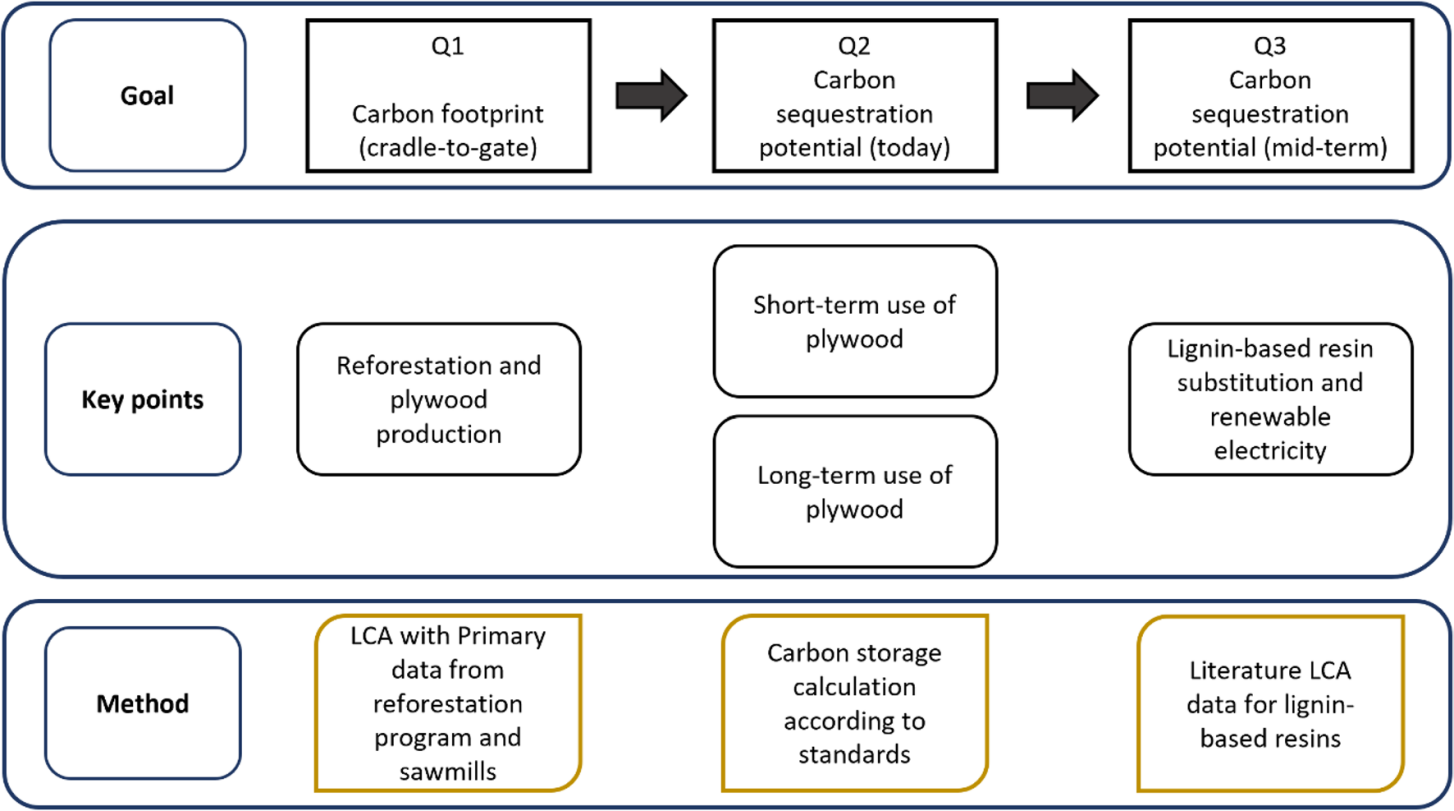
Structure of analysis with research questions Q1 to Q3.

A cradle-to-gate life cycle assessment (LCA) in accordance with ISO 14040^43^ and 14044^44^ is carried out using primary FVW data of reforestation and plywood production in Kalimantan (Fig. 1). This cradle-to-gate LCA is complemented of temporarily stored carbon in the plywood products using the carbon accounting standards ISO 14067 and PAS 2050/ILCD. Both short- and long-term storage in furniture and timber framing in house construction, respectively, are analyzed. Finally, effects from substituting current fossil-based with lignin-based resins are analyzed based on literature data.

### Cradle-to-gate life cycle assessment

#### Goal and scope

**Figure 2:**
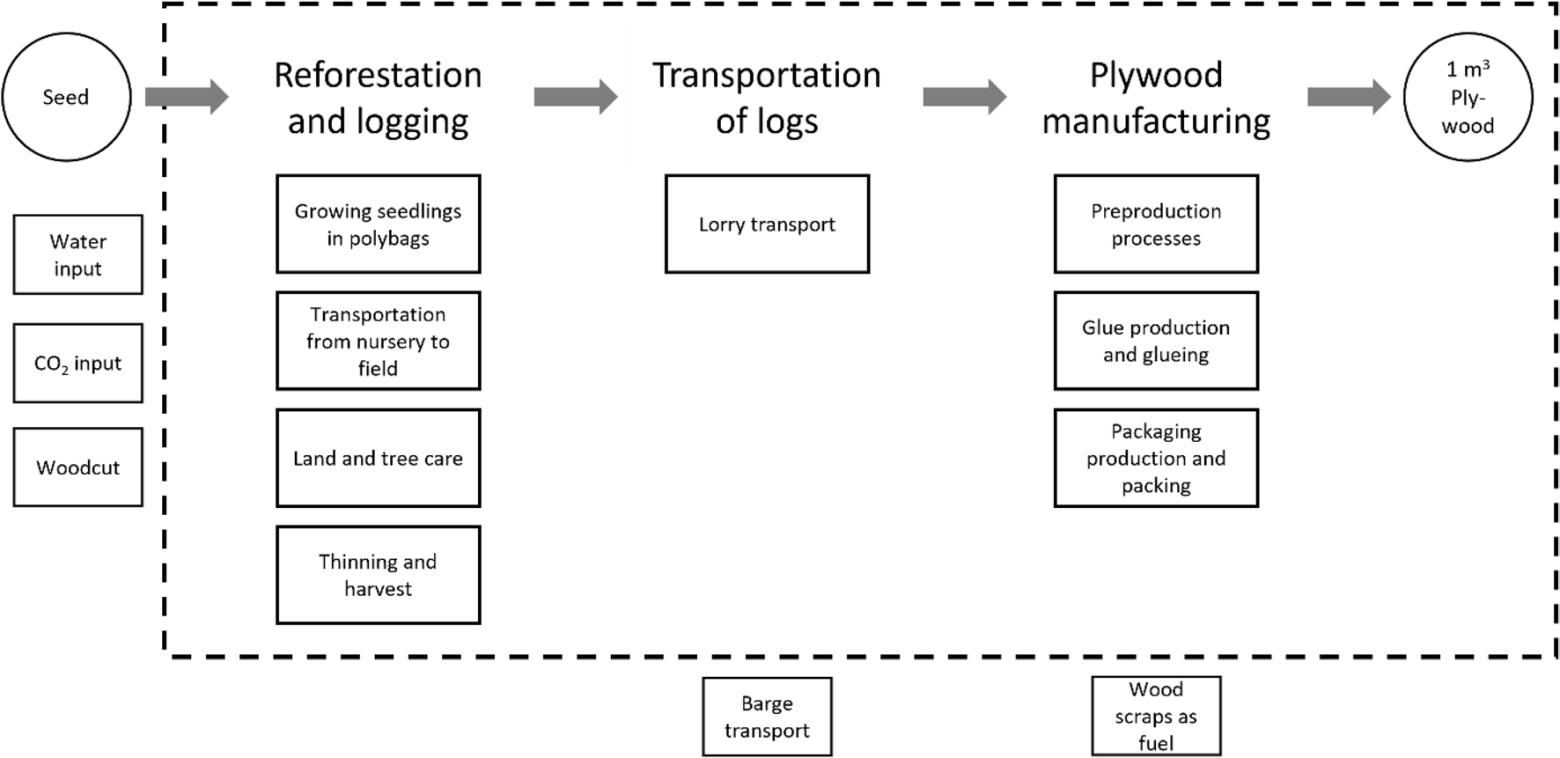
System boundary of the cradle-to-gate LCA model.

The goal of the cradle-to-gate LCA is to identify processes with the highest impacts on GHG emissions to identify potentials for improvement and to provide data for the subsequent carbon storage analysis. Only the phases reforestation and logging, transportation of wood logs, and plywood manufacturing are considered (Fig. 2). As declared unit 1 m^3^ packed plywood is used (instead of a functional unit acc. to ISO 14040/44, a declared unit acc. to ISO 14067 is used. This allows direct comparisons with other GHG studies on plywood.). The period under consideration is the 2019/2020 planting season.

### Life cycle inventory (processes)

For the reforestation and logging phase primary data from the fairventures project was used, provided by FVW as personal communication. Data for the transportation of logs and plywood manufacturing stem from three plywood manufacturing companies (sawmills) located in Central Java, who also process sengon logs from the fairventures project. The LCA was modeled with Umberto LCA +^45^ using ecoinvent 3.5 database^46^.

#### Reforestation and logging

Sengon seedlings are grown in polyethylene polybags in a media consisting of topsoil, compost, cocopeat, ricehusk and clay in a tree nursery in Kalimantan. As fertilizer, NPK-16-16-16, herbagreen® Z20 (cf.^47^) and herbagreen® protect F, both leaf fertilizers (cf.^48^), are used. The herabagreen® products were cut off. Machines for mixing the growing media and water pumps are diesel-fueled. After 9 weeks of growing, the seedlings are transported to the plantation with a pickup truck for which the diesel consumption is accounted for in the lifecycle inventory (LCI) model. Prior to planting, the land is cleared with motor scythes and planting holes are prepared with an auger. The planted trees are fertilized with NPK, dolomite, biochar, compost and chicken dung. For an improved growth, the ground is weeded several times with motor scythes. Before the final harvest, the sengon trees are thinned with power saws two times for which gasoline and lubrication oil consumption are modelled. The harvesting is supported by tractors with a winch, that consume diesel. Logs are transported from Kalimantan to three plywood sawmills in central Java. The average distance from the plantations to these mills is 591 km.

#### Plywood manufacturing

Since also other wood products are produced in the three sawmills, electricity, fuel gas and other materials were physically allocated to the sengon plywood output. The arithmetic mean from all three sawmills was used for the LCA. A detailed overview of the typical production steps is provided by Hughes^35^. All three sawmills use urea–formaldehyde (UF) and melamine–formaldehyde (MF) resins. The hardeners were cut-off. The sengon plywood is packed with paper, cardboard, plastic wrapping, plastic strings and corners for which literature data by Rüter and Diedrichs^49^ was used.

### Carbon storage and delayed emissions

#### Carbon storage in plywood

Stored carbon is calculated according to Eq. (1):

1 $${\mathrm{P}}_{{\mathrm{CO}}_{2}}=\frac{44}{12}\times \mathrm{cf}\times \frac{{\uprho }_{\upomega }\times {\mathrm{V}}_{\upomega }}{1+\frac{\upomega }{100}}$$

Equation (1)*: Potential carbon dioxide emissions from oxidation of stored biogenic carbon in wood products according to DIN EN 16449*^50^*.*

*P*_*CO2*_ are potential carbon dioxide emissions of a wood product due to oxidation, *cf* is the carbon content in the dry biomass (a standard value of 0.5 was used), *ω* the remaining moisture in the product (8–12 percent for sengon wood^51^), *ρ*_*ω*_ the raw density of wood (350 kg/m^3^ ± 10% for sengon wood^51^), *V*_*ω*_ product volumes at moisture *ω*, adapted to the functional unit of 1 m^3^. Published values for moisture and raw density of sengon wood vary considerably (Table 1). The corresponding values for P_CO2_ calculated using Eq. (1) range from 421.94 to 916.81 kg CO_2_/m^3^ with an arithmetic mean of 669.38 kg CO_2_/m^3^. For plywood, the three sengon plywood mills use on average 10.13 percent resin per m^3^ plywood. This results in a carbon dioxide uptake of 602 kg CO_2_ per m^3^ sengon plywood.

**Table 1:** Literature values for sengon wood moisture and raw density. Literature^52^ and^53^ were cited in^28^.

Moisture [%]	Raw density* ρ*_*ω*_ [kg/m^3^]	Data source
8–12	350 ± 10%	^51^
12 ± 2	317	^30^
15	240–490	^52^
12	230–500	^53^

#### Biogenic carbon and delayed emissions in ISO 14067, PAS2050 and ILCD

Biogenic carbon, and emissions occurring when a product get oxidized after its use phase (so-called delayed emissions), are differently accounted for in the carbon accounting and LCA standards ISO 14067, PAS 2050, and ILCD. ISO 14067 requires to report carbon stored in the product separately and to include all biogenic GHG emissions^22^. Consequently, also GHG emissions arising from wood incineration during production have to be included. However, since the system under consideration is a reforestation project on degraded land, i.e. wood carbon was fixed only a few years prior to its release and no additional GHG emissions due to land use changes are expected, GHG emissions would be distorted. According to PAS 2050, carbon storage and delayed emissions can be accounted for in the CF calculation (cf.^23^ Annex E). PAS 2050 and ILCD are congruent here. If carbon is stored for at least 100 years, it is considered as not released. Therefore, two scenarios are considered in this study: i) plywood is used for furniture or as non-structural building material with a relatively short lifetime (definitely less than 100 years) and ii) plywood is used as a structural building material and remains unoxidized for more than 100 years. In the former case, it is assumed that the materials are thermally recovered such that the carbon fixed in wood is re-emitted in the 100 years. According to the standards above, this emission is directly accounted for and no credit for delayed emissions is provided. In reality, one would expect a mix between long-term and short-term plywood use and a CF that lies between the two extremes.

### Lignin-based adhesives for plywood

**Figure 3:**
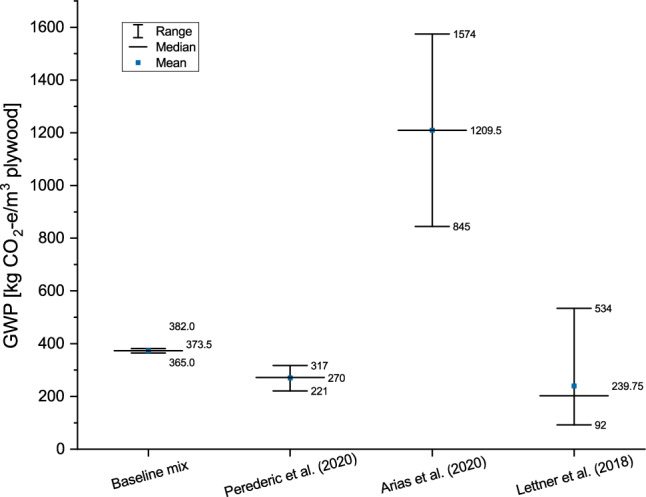
Calculated GWP of fossil-based resin (baseline mix) in comparison to GWP values of lignin-based resins in literature (all data for 1 m^3^ plywood).

Whereas GHG emissions of the fossil-based MF and UF adhesives were modelled using primary data from the local sawmills about the mixtures applied and ecoinvent data for their production, for lignin-based adhesives LCA data from Perederic et al.^55^, Arias et al.^41^ and Lettner et al.^54^ were used. LCA results were converted to 101.3 kg of resin as reference flow, the mass needed for one m^3^ of plywood. Figure 3 shows the large ranges and differences (92.2 kg CO_2_-e/m^3^ in ^54^, 1574.2 kg CO_2_-e/m^3^ in^41^) in the studies. These arise from different types of lignin assessed (kraft lignin and organosolv lignin), differences in resin mixtures and system boundaries. Arithmetic means were calculated over all resin mixtures in the respective studies. Whereas all resin systems in Perederic et al.^55^ perform slightly better than the fossil baseline mix, the mixtures in Arias et al.^41^ and Lettner et al.^54^ show larger ranges, and those in Arias et al.^41^ show considerably higher GWP values.

The resins above do not only differ with respect to their GWP, but also in their technical properties. The glyoxalated lignin from^41^ has been tested in combination with tannin and hexamine in triple-layer plywood. Plywood boards made from pine and oak and from beech meet standard requirements for interior use^40^. Lettner et al. make no reference to papers describing the use of the two adhesives made from modified lignins for plywood. However, the three-layer plywood boards in Kouisni et al.^56^ are equally made from adhesives with phenol replaced by kraft lignin. Tests according to Canadian standards showed that not more than 30 percent of the phenol should be replaced by lignin^56^. Perederic et al.^55^ investigated organosolv-lignin resins with a weight share of lignin to phenol of 0:1 for phenol formaldehyde (PF), 2:3 for lignin‐based phenol formaldehyde (LPF) and 1:0 for lignin formaldehyde (LF) resin in a cradle-to-gate study. No mechanical tests were carried out on the stability and standard conformity of the resins. However, the adhesives are comparable to those in Tachon et al.^42^, which meet according to these authors the requirements for indoor use according to standard NF EN 314–1.

Thus, it can be summarized for the lignin-based adhesives, that plywood manufactured with these adhesives is at least suitable for indoor use, e.g., for building structure, although the water resistance of bio-based adhesives remains a challenge^57^.

## Results

### Cradle-to-gate LCA

**Figure 4:**
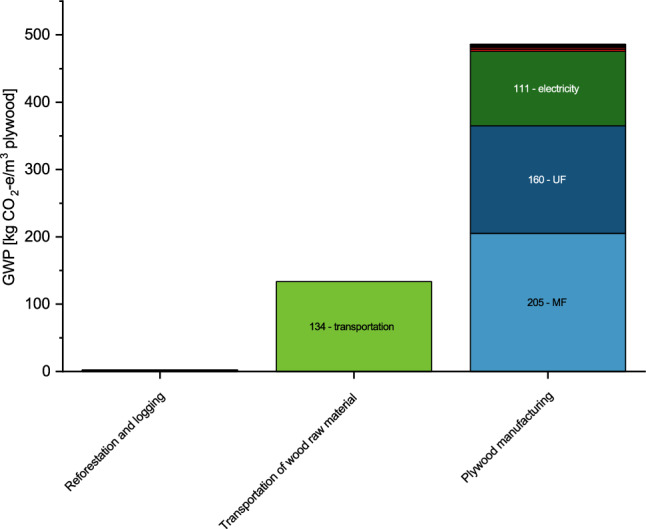
Cradle-to-gate LCA: GWP (100 years) results for plywood from the FVW social reforestation program (MF for melamine–formaldehyde and UF for urea–formaldehyde resins).

Cradle-to-gate GHG emissions for plywood amount to 622 kg CO_2_-e/m^3^. GHG emissions of reforestation are negligible compared to those from the transportation of logs and plywood production (Fig. 4). Whereas 18% of total GHG emissions can be attributed to the generation of electricity used in plywood production and 21% to transportation of logs, 59% result from the production and application of resins.

### Scenarios for delayed carbon emissions

Plywood use for furniture or as non-structural building material with a shorter lifetime than 100 years (scenario one) results, according to the standards ISO 14067, PAS 2050 and ILCD, in GHG emissions of 622 kg CO_2_-e/m^3^. In case of long-term carbon storage in structural construction material (scenario two), GWP is—if PAS 2050 or ILCD are followed—reduced by the carbon stored above 100 years which results in a CF of only 21 kg CO_2_-e/m^3^. Note that this reduction wouldn’t have been provided if the plywood had its origin from a virgin forest. Depending on the share between plywood use in products with short and long (above 100 years) lifetime, the CF varies between 622 kg CO_2_-e/m^3^a (only use in products with short lifetime) and 22 kg CO_2_-e/m^3^ (only use in products with > 100 years lifetime).

### Lignin-based adhesives and electricity produced from renewables

**Figure 5:**
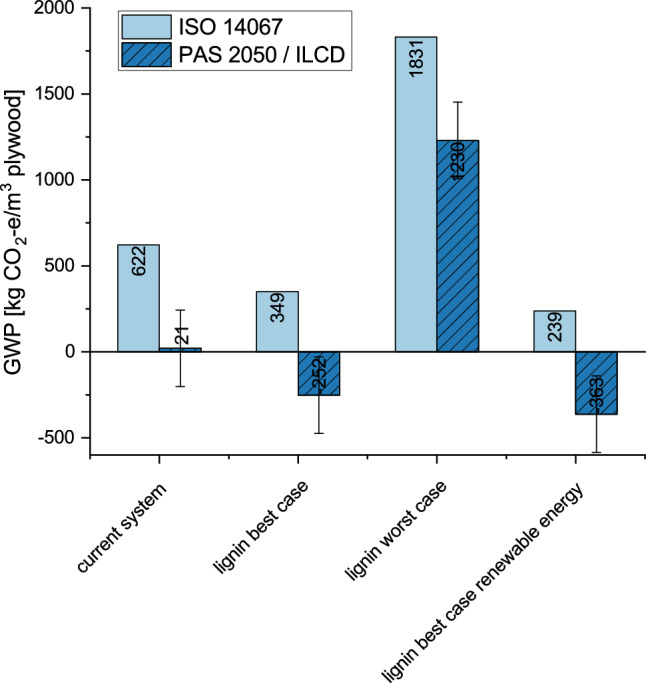
GWP of plywood systems (only systems with product lifetime > 100 years) with fossil-based adhesives (current system/baseline) and substitution by lignin-based adhesives according to standards ISO 14067 and PAS 2050/ILCD, all systems with Indonesian electricity mix, except for system on the right for which renewable electricity was assumed.

The effect that a substitution of the fossil-based adhesive with different lignin-based adhesives ones would have on GWP, is shown in Fig. 5 for the scenario with long-term (> 100 years) storage in products. To show the spread, only lignin-resins with lowest and highest GWP are presented. The last two bars on the right show that a switch from the current electricity mix in Indonesia to one based on renewables could reduce GWP by 110 kg CO_2_-e/m^3^.

## Discussion

The results of the cradle-to-gate LCA show that independent of the carbon accounting standards applied, small net positive GHG emissions will occur if wood from the FVW social afforestation program in Indonesia is used in plywood with lifetimes above 100 years. Here, it must be kept in mind, that due to the uncertainties, some carbon sinks such as build-up of soil organic matter in agroforestry are not accounted for. In their meta-analysis, Shi et al.^58^ show that in agroforestry systems significantly more carbon is stored in soils than in usual cropland. Agroforestry may also have positive effects on soil quality of degraded lands^5,58^. Additional manual accounting for land-use changes is not trivial and could lead to double-counting due to LCI datasets that already have LUC included^59^. Furthermore, material substitution effects were neglected in the LCA. The additional supply of plywood will replace at least to some extent other materials. A part of the supply might also induce additional demand. Especially in the building sector, the use of bio-based materials instead of emission-intensive materials such as cement can save large amounts of GHG^60^. Further, side products of veneer and plywood production such as saw dust and wood cut were incinerated for heat and electricity production. If heat and electricity replace the combustion of fossil fuels outside of the LCA system boundary, a credit could be provided for this^25^. In the case study, however, a conservative approach was selected, and no credits were given. Overall, the calculated GWP for sengon plywood is with 622 CO_2_-e/m^3^ of similar size as that of meranti plywood with a range of 329 to 592 kg CO_2_-e/m^3^ from Indonesian and Malaysian production with an arithmetic mean of 446 kg CO_2_-e/m^3^ ^36^. Compared to the production of plywood with UF resin in Germany, which according to Rüter and Diedrichs^49^ only has emissions of 266 kg CO_2_-e/m^3^ for the production and transport phase, the emissions are very high. This is partly due to the fact that the German electricity mix contains a significantly higher share of renewable energies (~ 40%)^61^ compared to Indonesia (~ 6%)^62^. Literature data on the density of sengon wood is used to calculate the carbon stored in the wood. The densities range between 230 and 500 kg/m^3^. The density has a particular impact on the calculation of GHG emissions in Fig. 5 (see error bars), as here the emissions calculated according to PAS 2050/ILCD also include biogenic emissions and storage in the product.

A problem with biogenic carbon emissions in LCA and carbon accounting is, that there is no scientific consensus how to deal with it^63^. This is especially true for temporally stored biogenic carbon^64^. Hoxha et al.^65^ recommend dynamic biogenic carbon accounting which is in accordance with the ILCD Handbook^66^. The disadvantage of dynamic accounting is the increased complexity and limited applicability for this study. Since the project under study is still rather in its early stages, information about the fate of produced plywood is lacking. Consequently, two scenarios for the use-phase of the plywood were developed: i) plywood use in furniture and ii) use for structural housing elements. Besides, cascade use of wood is possible and would affect the LCA results (e.g.^67^). However, recycling of plywood is still demanding due to the UF resins^68^ and even more if the wood is contaminated with building materials and wood preservatives^69^.

The mixture of fossil-based UF and MF resins is responsible for a large share of GHG emissions of the plywood. Bushi et al.^70^ conducted an LCA for North American resins for wood processing and found that extraction of the raw materials and the upstream processes cause by far the largest share of GHG emissions, 91% for UF and 92% for MUF. But lignin-based adhesives are already industrially used, for example, by the plywood manufacturer Latvijas Finieris. According to their plywood handbook^71^, the company produces various birch plywood boards of which one, a double-sided sanded birch plywood, also meets the requirements for bonding quality of EN 314-2^72^ when glued with lignin-based adhesive and can be used indoors and outdoors, for example for transport, packaging and children’s toys^73^. Resin substitution with lignin-based resins is not yet implemented in the project area in Indonesia and thus modelled based on literature data. To implement the value chain as locally as possible and to avoid emissions from long transport routes, lignin should also be produced regionally. Today, a technology readiness level (TRL) of 4 is assumed for lignin production in Southeast Asia^74^. Another limitation in the use of lignin-based resins is their higher cost compared to fossil-based resins. Both cost and performance are strongly positively correlated with the number of modifications and the functionalization of lignin^75^. At the same time, unmodified lignins are not suitable for use in wood adhesives^76^. So, it is a typical trade-off between properties and costs. However, it can be assumed that due to increasing prices on GHG emissions, as well as promotion programs for the use of renewable raw materials, and the general technical progress in the field of bio-based chemicals, the costs of lignin-based adhesives can keep up with fossil-based adhesives in the medium to long term.

## Conclusion

A social reforestation program in Kalimantan, Indonesia, helps rural farmers to establish an agroforestry system on degraded uncultivated soil such that the local population can supply itself with food and income from selling wood. Six years after planting, the trees are harvested and sold to sawmills in Kalimantan and Java. The main product from those sengon logs is plywood.

To evaluate if carbon can be sequestrated this way, GWP of wood production was determined using primary data from the reforestation and logging processes and plywood production making use of data collected in a survey of three sawmills that process sengon logs to plywood. Total GHG emissions sum up to 622 kg CO_2_-e/m^3^ plywood of which 59% originate from the production and application of resins. In a scenario analysis the effects of biogenic carbon storage in plywood products on GWP were assessed. According to the carbon accounting standards ISO 14067, PAS 2050 and ILCD, storage time below 100 years, for which uses for furniture or for non-structural parts of buildings could serve as example, will not alter the GWP of 622 kg CO_2_-e/m^3^. However, if plywood remains unoxidized for more than 100 years, which could be the case if plywood is used as structural element in construction, GWP is reduced to 21 kg CO_2_-e/m^3^. The one or the other GWP needs to be compared with the GWP of other materials which would be replaced by the plywood such as plastics, concrete, or steel, depending on the application case.

The strong impact of the fossil-based resins on total GWP was starting point for a literature review to identify suitable alternatives. As lignin is the second most common biopolymer in nature and could be obtained from the wood residues of sengon log production and at the same time is similar to the widely used phenolic resin due to its chemical structure as a polyphenolic polymer, current LCAs of lignin resin production were evaluated. The lignin-based adhesives not only vary in their technical properties but also in their GWP. In fact, large differences result from different adhesive compositions and manufacturing processes. If the highest reported value for lignin-based adhesives^41^ would be taken, GWP would almost triple from 622 kg CO_2_-e/m^3^ to 1831 kg CO_2_/m^3^, while for the lowest reported value^54^ GWP would be almost halved to 349 kg CO_2_-e/m^3^. In case that the plywood would remain unoxidized for more than a 100 years and PAS 2050 would be applied as carbon accounting standard, the GWP would become even strongly negative with − 252 kg CO_2_/m^3^. In summary, the currently produced plywood from the reforestation program likely do not have a sequestration effect, unless long-lasting products with lifetimes of more than 100 years are made from it. Note that build-up of soil organic matter and the associated carbon sequestration were neglected here due to a lack of data. Room for improvement is when switching to electricity from renewable energy in plywood production. This alone could decrease the GWP of plywood by about 110 kg CO_2_/m^3^, i.e. by one sixth in case of fossil-based adhesives to one third in case of the lignin-based adhesive with the lowest GWP. When looking at the pure GWP values, one should also keep in mind that even storage for a few decades is already beneficial for climate change mitigation, even if it is not accountable according to carbon accounting standards. Even more, the plywood and food produced on previously uncultivated land reduces pressure on virgin forests and thus also deforestation. Deforestation may have a strong impact on GWP of a product as shown by Nguyen et al^77^ who report that the GWP of beef from Brazil is higher by a factor of 3.1 to 3.9 if land use changes are accounted for and depreciated over 20 years. Method-wise such an analysis would require a consequential LCA and is a topic for further research.

Future research should also investigate the technical and environmental performance of sengon plywood with lignin-based adhesives and its application areas. Furthermore, effects on emissions of GHG other than CO_2_, e.g., N_2_O or CH_4_ from uncultivated land vs. land under agroforestry, needs to be assessed. Finally, long-term effects of agroforestry with sengon plantations need to be assessed, also the stability of the system. Overall, the social reforestation program is expected to have a number of benefits for the local community with the additional potential to contribute to climate change mitigation in the mid-term.

## Data Availability

The datasets generated during and/or analyzed during the current study are available from the corresponding author on reasonable request, provided that no copy rights are violated.
